# Properties of bundle valuations in carrier collaboration

**DOI:** 10.1007/s10100-023-00873-1

**Published:** 2023-07-26

**Authors:** Rudolf Vetschera, Dmitriy Knyazev, Daniel Rehsmann

**Affiliations:** Department of Business Decisions and Analytics, University of Vienna, Oskar Morgenstern Platz 1, 1090 Vienna, Austria

**Keywords:** Carrier collaboration, Combinatorial auction, Monotonicity, Additivity, TSP

## Abstract

Combinatorial auctions are an important part of many frameworks for carrier collaboration in logistics. In the last years, many innovative auction mechanisms have been developed in the economic literature that could potentially be applied in this context and that could significantly simplify the process of determining efficient allocations of requests. These mechanisms rely on certain assumptions about the valuations of bidders. In this paper, we perform computational experiments to analyze to which extent these requirements are fulfilled for valuations derived from a simple logistics problem, the Traveling Salesman Problem. We find that bidders’ valuations typically violate even very elementary requirements, but that these violations affect only a limited number of request bundles. We further analyze whether these violations exhibit a consistent pattern across multiple carriers and check consistency if route lengths are calculated exactly or approximated.

**Supplementary Information:**

The online version contains supplementary material available at 10.1007/s10100-023-00873-1.

## Introduction

Carrier collaboration has become an essential topic in logistics research (see, e.g., Gansterer and Hartl 2018b; Speranza 2018). By exchanging transportation requests among each other, logistics providers can increase the efficiency of the overall logistics system since requests can be performed by the carrier who can execute them with the least costs. Many frameworks for carrier collaboration have been proposed in literature (Krajewska and Kopfer 2006; Ergun et al. 2007; Gansterer and Hartl 2018b).

Although centralized approaches (commonly implemented via some mathematical programming model) remain a benchmark in terms of efficiency (see, e.g., Chen 2016; Berger and Bierwirth 2010), such models rely on the assumption that carriers are willing to reveal confidential information. Given this issue, the dominant approach for carrier collaboration utilizes decentralized and information-preserving frameworks, at the core of which is some form of combinatorial auction. Various routing problems have been tackled using an auction as a post-market exchange. A traveling salesman problem with precedence constraints is discussed in Berger and Bierwirth (2010), highlighting the potential of auction-based exchange mechanisms over individual control and planning, provided that information is shared truthfully within auctions. Relaxing the assumption on non-strategic carriers, Figliozzi (2006) simulates the performance of incentive-compatible exchange mechanisms in the context of truckload pickup-and-delivery services with time windows. Karaenke et al. (2019) discuss a one-to-many slot management model, where incentive-compatible auction mechanisms are deployed to coordinate slots among several carriers. An exchange mechanism to allocate multi-attribute units in the context of transportation procurement is proposed by Zhang et al. (2019).

Two-sided markets in less-than-truckload transportation are studied in Chen (2016), allowing carriers to take on hybrid roles (sellers and buyers) by utilizing the clock-proxy auction proposed by Ausubel et al. (2017). The issue of bilateral exchange is further analyzed in the setting of truckload carrier collaboration by Xu et al. (2017), which provide incentive compatible mechanisms based on the well-known trade reduction mechanism of McAfee (1992), allowing also multiple-unit demand. The complexity of large-scale problems is tackled, among others, by Gansterer and Hartl (2018a); Rüther and Rieck (2022) addressing the issue of optimal bundle generation for combinatorial auctions and Mancini and Gansterer (2022) in the context of last-mile delivery with occasional drivers. Triki et al. (2014) provide an alternative approach to large scale problems by integrating bid- and price generation with the routing of truck-load carriers. A framework for the exchange of a single request is embedded in Li et al. (2015). Alternative collaboration approaches to auctions are discussed, among others, in Guajardo and Rönnqvist (2015) on the basis of coalition formation and Guajardo and Rönnqvist (2016) based on efficient cost allocation. The common understanding of the literature is that since the costs of performing a request strongly depend on the other requests a carrier has to serve, requests cannot be evaluated individually. Thus, carriers need to know the entire set of requests they could receive when bidding in an auction.

In parallel to the literature on carrier collaboration, research on combinatorial auctions has also made considerable progress in recent years. Many new auction designs have been proposed that provide efficient allocations and incentive compatibility (i.e., to incentivize bidders to state their true valuations). Most of these mechanisms rely on specific assumptions on the properties of bidders’ valuations of the items to be allocated, such as monotonicity or superadditivity (definitions of these properties will be given in Sect. 3). To make these results available for the design of auction mechanisms for carrier collaboration, verifying whether their underlying assumptions are fulfilled in the case of transportation requests is necessary. However, to our knowledge, this question has not yet been addressed in the literature.

Therefore, this paper aims to study whether valuations of transportation requests by carriers fulfill critical assumptions on preferences required for innovative combinatorial auction mechanisms and to what extent these assumptions are fulfilled or violated. More specifically, we consider a setting where carriers already have to serve specific requests and are bidding for additional requests in an auction. Thus, we consider marginal evaluations of bundles of requests in addition to an already existing set of requests. Valuation in this context can be considered as the marginal costs of fulfilling the additional requests (in the case of a reverse auction where bids represent the compensation a bidder demands to take over a bundle of requests). Alternatively, valuations could refer to the marginal profit of a request if the contract is transferred to another carrier that receives the price a customer pays for fulfilling the request. In that case, the auction is a standard auction. The bid represents the amount the carrier obtaining the request is willing to pay to the carrier initially holding the request (or the auctioneer) to obtain the request and its associated revenue. In this paper, we will consider both possibilities.

Properties such as (super)additivity refer to the valuations of one bidder for bundles of items. For the design of auction mechanisms, it is also essential to know whether these properties are the same across all bidders participating in the auction. In a recent paper, Baldwin and Klemperer (2019) introduced the concept of *demand types* of bidders, which are characterized by the sets of items that are substitutes (exhibit negative synergies) or complements (exhibit positive synergies) for a bidder. They showed that a structural property of the participating bidders’ demand types, unimodularity, is a necessary and sufficient condition for the existence of a competitive equilibrium for arbitrary valuations. Thus, that property would significantly simplify the auction design because each request could be priced separately. Therefore, we extend our initial research question to study whether structural properties of valuations are the same or different among carriers.

Participation in a combinatorial auction requires that bidders evaluate a large number of possible bundles that they can bid for. However, to make a bid, it is not necessary to determine the solution of the underlying transportation problem (e.g., the actual route to be traveled); it is sufficient to know the costs and, thus, the optimal travel distance needed to fulfill these requests. Following this argument, approximation methods to estimate optimal tour lengths were developed (e.g., Nicola et al. 2019; Akkerman and Mes 2022; Kou et al. 2022). These methods can achieve high degrees of fit between actual optimal tour lengths and approximations, explaining over 90% of the variance in optimal tour length. Nevertheless, given the arguments presented above, it might be more important that such approximations preserve structural properties of valuations rather than closely correspond to numerical values. If, for example, valuations are close to additive, a small change in the valuation of a combined bundle might change a subadditive valuation into a superadditive one or vice versa. Our next main research question, thus, is whether structural properties of valuations generated using approximations of total tour lengths correspond to structural properties of valuations based on tour lengths resulting from the optimal solution of the underlying transportation problem.

We do not expect the answers to these research questions to be identical in all problem contexts. Factors like the number of requests to be allocated to carriers in relation to the requests they already have to serve or the geographical location of these requests most likely will influence whether structural properties are fulfilled. Our final research question thus deals with factors that might influence the answers to the previous two research questions.

We perform a computational study to answer these research questions. The context of these experiments is the Traveling Salesman Problem (TSP). There are several reasons for performing such experiments using structurally very simple problems such as the TSP rather than more realistic problems. The first is that to answer the second research question; we need exact solutions to the underlying problem, which can be obtained efficiently for the TSP, while one would have to resort to heuristics for more complex problems. Secondly, suppose we can show that structural properties are violated already in the TSP. In that case, it is quite clear that they will also be violated, possibly to a larger extent, in more complex problems. Thus, analysis of the TSP provides a good starting point before moving on to more complex problems if necessary.

The remainder of this paper is structured as follows. Section 2 reviews recently proposed auction mechanisms. In Sect. 3, we provide exact definitions of the properties studied in this paper, show their importance for auction design, and give some theoretical arguments on why we can expect these conditions to be fulfilled or violated in the context of transportation requests. Section 4 introduces the design of the computational study and the model used. Simulation results are presented in Sect. 5, and Sect. 6 concludes the paper by discussing the results and providing an outlook on future research.

## Auction mechanisms

Auctions are widely used mechanisms for discovering market-clearing prices. One of the most prominent theoretical concepts is the VCG auction introduced by Vickrey (1961), Clarke (1971), and Groves (1973). In this auction, bidders must pay a price equal to the externality they impose on other bidders. This auction satisfies two key requirements. First, it always provides an efficient allocation. Second, it is dominant-strategy incentive compatible, meaning it is a dominant strategy for bidders to report their valuations truthfully. These valuable features hold both for the cases of divisible and indivisible goods and any preferences of bidders: complements, substitutes, and mixtures thereof. Although theoretically an “ideal” mechanism, it is almost impossible to implement in practice. In a VCG auction, bidders have to submit their preferences over any possible bundle of goods, making this auction extraordinarily costly and time-consuming to implement. For example, even with 20 items, each bidder must submit $2^{20}$ $=$ 1,048,576 valuations. Furthermore, it requires the solution of several allocation problems resulting from dropping each bidder in turn. Moreover, based on the bidder’s valuation vectors, the auctioneer has to determine the welfare-efficient partitions, a maximization problem whose complexity increases exponentially. This gives rise to one of the major criticisms of the VCG approach, i.e., the lack of a competitive price system. Practically, it asks all bidders to reveal all privately known valuations to the auctioneer directly. In the application context of private companies such as carriers, bidders are likely reluctant to expose sensitive information. In practice, computationally simpler and informationally efficient auctions must be used.

Often these auctions take the form of some tâtonnement process, which works roughly as follows. First, the auctioneer announces a price vector. Then, bidders report their demand vectors at these prices. Given some excess demand (supply), the auctioneer increases (decreases) components of the price vector. The crucial question in applying such tâtonnement processes is whether it converges to a Walrasian equilibrium. A Walrasian equilibrium is a situation such that there exist linear prices (i.e., prices of bundles are the sum of prices of their components) so that there is no overdemand or oversupply of any items. Arrow and Hurwicz (1958) and Arrow et al. (1959) show that tâtonnement processes converge to equilibrium when goods are divisible and have *price substitutes* property. However, Scarf (1960) shows that when goods are *price complements*, there may be no convergence to equilibrium even with divisible goods. In economic theory, goods are considered substitutes if increasing the price of one good increases the demand for the other good and complements when it decreases the demand for the other good.

In the case of transportation and logistics problems, the goods are indivisible, further complicating the problem. Kelso and Crawford (1982) show that for indivisible goods, the equilibrium may not exist at all. They introduced a crucial property called *gross substitutes* (GS) that ensures the existence of a Walrasian equilibrium for economies with indivisible goods. Sun and Yang (2006) generalize the *GS* property to what they call *gross substitutes and complements* (GSC). This condition essentially requires that all items can be divided into two groups of goods such that the items within one group are substitutes and between the groups are complements.

Building on these concepts, several dynamic auctions have been proposed that rely on the *GS* property. Gul and Stacchetti (2000) were among the first to develop a dynamic auction to sell indivisible items that assumes both *monotonicity* and *GS* properties. In a seminal paper, Ausubel (2006) presents a dynamic auction that yields an efficient outcome, Walrasian equilibrium price vector, and sincere bidding in equilibrium. For the case of divisible goods, the only conditions needed for that are private values, concave quasilinear utility, and *monotonicity*. In the case of indivisible goods, a mechanism developed by Ausubel (2006) requires that goods also exhibit the *GS* property. Sun and Yang (2009) propose a dynamic incentive-compatible auction that results in a Walrasian equilibrium when goods satisfy the *GSC* property.

In carrier collaboration, the requests may not exhibit *gross substitutes* or *gross substitutes and complements* properties because, firstly, requests with close delivery destinations may be complements, thus violating the *GS* condition. Secondly, two carriers might perceive transportation requests differently; while for one carrier, two requests are complements, a different carrier may perceive them as substitutes, thus violating the *GSC* condition. Therefore, the existence of an equilibrium is not guaranteed in this case. Hence, some auctions with non-linear prices need to be considered for carrier collaboration.

Ausubel and Milgrom (2002) propose a dynamic auction for a situation where bidders have private values, quasilinear utility, and the seller has zero values for all goods. On top of that, the crucial property required is *monotonicity*. They propose an auction in the spirit of the deferred acceptance algorithm first introduced in Gale and Shapley (1962), which is very popular in the matching literature. An adaptation of this algorithm to auctions works as follows. In each round, bidders can make mutually exclusive bids on any bundles. A bidder cannot reduce or withdraw any bid, and any new bid on some bundle must exceed the bidder’s previous bid on that bundle. After each round, the auctioneer announces the “currently winning" bids by choosing the set of bids that maximizes the total revenue conditional on each bidder “currently winning" at most one bid. Once there is a round in which any bidder submits no new bids, the auction stops. The “currently winning" bids at that round become the actually winning bids. Ausubel and Milgrom (2002) show that if bidders bid truthfully, the outcome of such an auction lies in the core concerning the true preferences; that is, the seller cannot do better by canceling the auction and negotiating with any of the bidders.

The drawback of the mechanism by Ausubel and Milgrom (2002) is that the prices that bidders need to pay for the bundles are not only non-linear but also discriminatory (i.e., they are different between bidders). Since discrimination is not accepted in many real-life situations, some non-discriminatory procedure is needed. Sun and Yang (2014) is the first paper that concentrates solely on complementary items. They propose a dynamic incentive-compatible auction that results in an efficient allocation when all bidders have private values, quasilinear utility, and *superadditive* valuations.

Generally, we see that the monotonicity of goods and complementarity/substitutability are crucial properties to make many auction formats work. It is also well known that *superadditivity* is a stronger condition than complementarity (e.g., see Lehmann et al. (2006)). Thus, as long as the valuations are *superadditive*, auctions that rely on the *GS* assumption may not work.

## Properties

This section discusses commonly posed restrictions on valuations used in prominent action designs. In light of the transportation applications we have in mind, we pay special attention to indivisibilities, particularly for heterogenous components that might exhibit synergies. We introduce our notation used throughout the paper for the case of indivisible goods and provide related assumptions on valuation vectors.

A finite set of market participants *I* wishes to exchange a set $N=\{1,2,\ldots ,n\}$ of heterogeneous indivisible goods. Every participant $i\in I$ attaches a monetary and integer-valued private valuation $v_i:2^N\rightarrow {\mathbb {Z}}_+$ to each bundle of items $S\in 2^N$, where the power set $2^N$ denotes the collection of bundles. We assume that market participants have quasilinear utilities, the utility of participant *i* from any bundle $S\in 2^N$ is $u_i(S)=v_i(S)-p(S)$, where $v_i(.)$ is the participant’s valuation and $p(\cdot )$ denotes the price specified for a specific exchange.

We study the structure of the interdependence between items of a certain bundle based on individual valuations, i.e., we discuss restrictions based on binary relations denoted by $\sim$ over subsets $v_i(S)\in V_i(2^N)$, representing individual valuations. To discuss the consistency of valuations among bidders, we explore relations like $V_i(2^N) \sim V_j(2^N)$ for any $i,j\in I$ in a separate section. Synergies or the interferences of items are framed by specifying binary relations between sets of bundles. A condition widely used in the literature to describe items with interferences requires the bidders to have gross substitutes (GS) valuations. A market participant views two bundles as GS if, whenever the price of one bundle *A* increases and the price of a different bundle *B* remains constant, the demand for *B* increases. A result due to Murota (2018) allows us to investigate such valuations without specifying price vectors since they establish the equivalence of GS valuations with the following condition, introduced by Gul and Stacchetti (1999).

### Definition 1

(Strong no complementarity (SNC)). The valuations satisfy the SNC condition, if and only if $\forall i \in I$, $\forall S_1,S_2\in 2^N$, and $\forall S_1'\subseteq S_1$ it holds, that $\exists S_2'\subset S_2$, such that

1 $$\begin{aligned} v_i(S_1)+v_i(S_2)\le v_i\left( S_1\setminus S_1'\cup S_2'\right) + v_i\left( S_2\setminus S_2'\cup S_1'\right) . \end{aligned}$$

We discuss the case of synergies between items following Sun and Yang (2014), in its most general notion, subsuming the case of gross complements and valuations based on the concept of supermodularity.^1^

### Definition 2

(Superadditivity (SA)). The valuations satisfy the SA condition, if and only if for any disjoint sets $S_1, S_2 \in 2^N$, every agent’s valuation satisfies

2 $$\begin{aligned} v_i(S_1)+v_i(S_2)\le v_i(S_1\cup S_2). \end{aligned}$$

Sub-additive valuations are defined accordingly, by reversing the inequality. A somehow intermediate case between gross substitutes and superadditive valuations follows the restriction on monotonic valuations by Ausubel (2006):

### Definition 3

(Monotonicity (M)). The valuations satisfy the M condition, if and only if for any $S_1, S_2 \in 2^N$, every agent’s valuation satisfies

3 $$\begin{aligned} {\text {max}}\{v_i(S_1),v_i(S_2)\}\le v_i(S_1\cup S_2) \end{aligned}$$

**Fig. 1:**
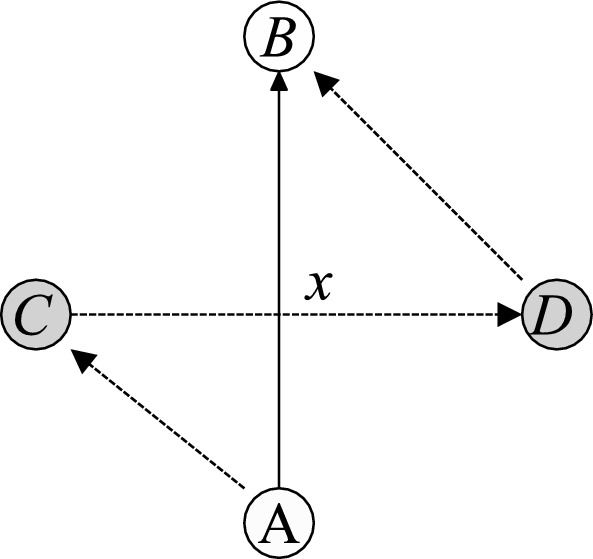
Example of a violation of superadditivity in a routing problem

Although one might intuitively assume that travel distances are always subadditive, and therefore valuations based on variable distance costs are always superadditive, this is not necessarily the case when considering marginal values. Consider the example shown in Fig. 1. A carrier has to serve requests *A* and *B*, and considers adding requests *C* and/or *D*. Adding only request *C* would require to travel distances *AC* and *CB*, but eliminates the need to travel *AB*. Therefore, the marginal effect of adding request *C* is $AC+CB-AB$. Similarly, the marginal value of adding request *D* is $AD+DB-AB$. If both requests are added, it is not necessary to travel the distance *AB*, but one needs to add $AC+CD+DB$ (or $AD+DC+CB$, for simplicity, we assume that the former is shorter). The sum of marginal values of adding the two requests individually is $AC+CB+AD+DB-2AB$. Subadditivity of distances (and thus superadditivity of values) would be violated if $AC+CD+DB-AB > AC+CB+AD+DB-2AB$ or $CD+AB > AD+CB$. Splitting the distances *AB* and *CD* at point *x*, this can be rewritten as $(Ax+xD) + (Cx+xB) > AD + CB$, which is clearly fulfilled because of the triangle inequality. Thus, the marginal travel distance resulting from both requests, in that case, is larger than the sum of the marginal distances of adding each request individually.

In contrast to the assumption of superadditive valuations, route lengths will always satisfy monotonicity due to the triangle inequality. However, this is no longer true when valuations of requests are based on profit. If the revenue associated with a request is given, then monotonicity will be fulfilled if the marginal cost of serving the request is lower than the request’s revenue and will be violated if marginal costs exceed revenue.

In the next section, we introduce our simulation model and check whether gross-substitutability, superadditivity, or the intermediate case of monotonicity holds in a TSP problem.

## Simulation model

The simulation model used for this study considered a setting in which carriers already have a set of requests and can bid for additional requests in an auction. As already indicated, we consider a TSP in this paper, so a request corresponds to a customer location to be visited once.

We expect that both the number of existing and additional requests and their location affects the occurrence of violations of monotonicity, additivity, and strong no complementarity (the SNC condition). Therefore, both factors were systematically varied in the simulation. Since the analysis requires the exact solution of a TSP, including all requests that a carrier already has plus all the additional requests, we limited the total number of requests to 12 and used situations with 4, 6, and 8 existing requests and thus evaluated bundles of up to 8, 6, and 4 additional requests. Hence, at most $2^8-1 = 255$ bundles had to be evaluated by determining the optimal route in a TSP with up to 12 customers.

**Fig. 2:**
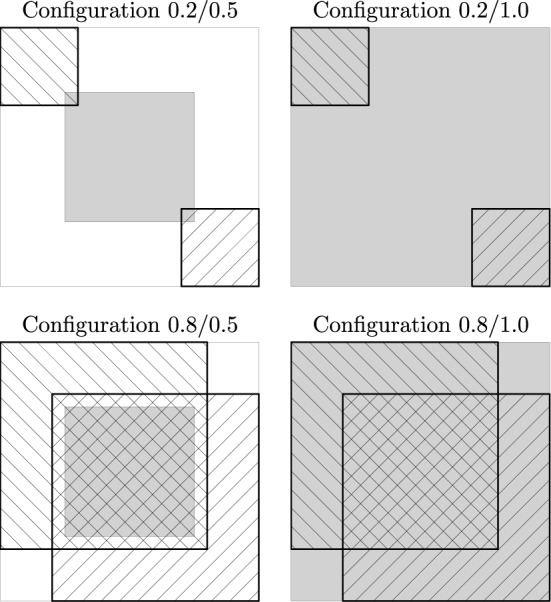
Configurations of geographical distributions of requests used in the simulation

Since we also want to study whether evaluations exhibit the same properties for different carriers, each set of additional requests was evaluated by two carriers with existing requests located in different areas. The entire area in which requests are located is the unit square. Each carrier has existing requests located at the top left part of the unit square for one carrier and the bottom right for the other carrier. To test different configurations, we set the sizes of the regions containing existing requests to 0.2 by 0.2 and to 0.8 by 0.8. Additional requests are located either in a region of 0.5 by 0.5 in the center of the unit square or the entire unit square. We identify the resulting geographical configurations of requests by indicating the size of the region of existing and the region of additional requests. The four configurations studied are shown in Fig. 2, where the two shaded areas indicate the regions in which existing requests of the two carriers are located, and the gray area indicates possible locations of additional requests. Coordinates of requests were generated as uniformly distributed random numbers in the respective areas.

We compare evaluations based on exact solutions of the underlying TSP and solutions obtained from approximating the optimal tour length. For the exact solutions, we used the Concorde solver (Applegate et al. 2001) via the R package TSP (Hahsler and Hornik 2007). Since Concorde requires all distances to be integer-valued, we used a square of size 10,000 by 10,000 to minimize the effect of rounding errors on distances.

The approximate solution was calculated using the approximation by Beardwood et al. (1959) as

4 $$\begin{aligned} d= \beta \sqrt{A \cdot C} \end{aligned}$$

where *C* is the number of customers to be visited, and *A* is the rectangle area containing the requests. Since we use tour lengths only to approximate costs, they can be arbitrarily scaled. We, therefore, set $\beta =1$ for simplicity.

With both approaches, we first calculated the (exact or approximated) length of a tour containing only the carrier’s existing requests and then the lengths of all tours containing the existing requests plus each possible bundle that can be formed out of the additional requests. We then used the difference between these tour lengths and the tour length without additional requests as the marginal costs of each bundle.

Since non-monotonic valuations are only possible for evaluations based on profit rather than costs, we also used several models for assigning revenues to requests. We assume that requests are priced individually, and thus the revenues of bundles are additive, i.e., the revenue of a bundle is the sum of the revenues of the requests contained in the bundle. Furthermore, we assume that carriers operate in a competitive market where the price for each request is sufficiently high so that at least one carrier (but not necessarily both) can fulfill every possible bundle of additional requests without incurring a loss.

We distinguish two scenarios for pricing requests. In the first scenario, prices are the same for all requests. We denote by $c_1(S)$ and $c_2(S)$ the marginal costs of carrier 1 (or 2) of serving all requests in bundle *S*. We then determine the price of each request as

5 $$\begin{aligned} p_{eq,one} = \max _{S \in 2^N} \frac{\min {(c_1(S),c_2(S))}}{|S|} \end{aligned}$$

when for each bundle, at least one carrier can cover its cost when fulfilling the request and

6 $$\begin{aligned} p_{eq,all} = \max _{S \in 2^N} \frac{\max {(c_1(S),c_2(S))}}{|S|} \end{aligned}$$

when assuming that for each bundle, both carriers can recover their costs. In the following section, we identify these two valuation models as *EqOne* (equal prices, non-negative profit of at least one player) for profits using pricing model (5) and *EqAll* for the other pricing model (6).

The second scenario considered different revenues for each request. We again assumed that the prices of bundles are additive. The prices $p_k$ for each request *k* were determined by solving the linear programming problem

7 $$\begin{aligned} \begin{array}{l} \max \sum _k p_k \\ \mathrm{s.t.}\\ \sum _{k \in S} p_k \le c(S) \; \forall S \in 2^N \end{array} \end{aligned}$$

where $c(S) = \min (c_1(S),c_2(S))$ for a pricing model in which at least one carrier can recover its costs for each bundle and $c(S)=\max (c_1(S),c_2(S))$ for a pricing model in which both carriers can recover their costs. We denote the valuation models as *DiffOne* and *DiffAll* to identify the differentiated pricing model. It should be noted that the profit that is calculated by forming the difference between this price and the costs (transportation distances) is still a valuation of a request by the bidder and that the bidder’s (quasilinear) utility is the difference between that valuation minus the price the bidder has to pay in an auction to obtain this request.

After generating evaluations of each bundle of requests using marginal distances or marginal profits according to the various pricing models, we test the resulting values for additivity and monotonicity. Let $v_i(S)$ be the valuation of bundle *S* based on one of the approaches described above. To test for monotonicity, we considered each request *k* and all bundles $S\in \{S: k \not \in S\}$ to which request *k* can still be added. Since there are *n* requests, in total,

8 $$\begin{aligned} n (2^{n-1}-1) \end{aligned}$$

such combinations are possible. We counted the number of cases in which the difference $v_i(S \cup \{k\}) - v_i(S)$ is negative, zero, or positive. A negative difference indicates a violation of monotonicity, and a zero difference indicates that request *k* has a marginal value of zero when added to bundle *S*.

Additivity was tested by comparing each bundle *S* to all bundles $T \in \{T: S \cap T = \emptyset \}$ and counting the instances in which the difference $v_i(S) +v_i(T) - v_i(S\cup T)$ is zero, positive, or negative. Note that positive synergies between bundles exist if that difference is positive for valuations based on distances and if it is negative for valuations based on profits. Since $$\left( \begin{array}{c}n\\ l\end{array}\right)$$ bundles of size *l* can be formed out of *n* requests, and for each of these bundles there are $2^{n-l} -1$ complementary bundles *T*, the total number of such comparisons is^2^

9 $$\begin{aligned} \frac{1}{2} \sum _{l=1}^{n-1} \left( \begin{array}{c}n\\ l \end{array} \right) (2^{n-l}-1). \end{aligned}$$

To test the SNC condition, we considered all pairs $(S_1, S_2)$ of bundles (including pairs of intersecting bundles). Since this condition involves an exchange of subsets of the bundles, it is only necessary to consider items for these subsets which are not contained in the other bundle, so the subsets $S_1'$ and $S_2'$ in condition (1) must fulfill the conditions $S_1' \subseteq S_1 {\setminus } S_2$ and $S_2' \subseteq S_2 {\setminus } S_1$. For each pair of bundles $(S_1, S_2)$, we generated all combinations of subsets $S_1'$ and $S_2'$ fulfilling these conditions and then tested whether condition (1) holds or not. Since this was computationally very resource intensive, we performed this analysis only for 250 (rather than 1000) problems in settings involving eight additional requests.

The simulation was programmed in R (R Core Team 2021) using packages lpSolve (Berkelaar et al. 2020) to solve the linear programming problem (7) and package TSP (Hahsler and Hornik 2007) to solve the TSP problems. We used a 3 (number of existing vs. added requests) $\times$ 4 (number of geographical configurations) complete factorial design. For each cell in this design, 1000 problems were randomly generated and evaluated using all 5 value models (Distances, as well as the pricing models *EqOne, EqAll, DiffOne, DiffAll*).

## Results

In this section, we discuss if the restrictions defined in Sect. 3 are satisfied in the simulation model defined in 4. We start with analyzing the imposed restrictions on the basis of individual valuations in Sect. 5.1. In the subsequent subsections, we discuss whether these structural properties are (i) consistent among carriers in Sect. 5.2 and (ii) consistent between exact and approximate calculations of distances in Sect. 5.3.

### Monotonicity, additivity and substitutability

Our first research question referred to the extent to which the conditions of monotonicity, additivity, and the SNC condition are fulfilled or violated in bundling transportation requests. We analyze this question at two levels: At the level of entire valuations, we consider the number of cases in our simulation in which the valuation of a bidder fulfills a condition for all pairs of bundles that could be generated. Subsequently, we consider the fraction of pairs of bundles for which the condition is fulfilled. While a violation in the case of one pair of bundles could be sufficient to let an auction fail, this is less likely to happen if only a few bundles are affected, so this measure provides an indication of how severely a condition is violated. Furthermore, in such a situation, it might be possible to exclude certain bundles.

**Table 1:** Fractions of problems (in %) in which monotonicity if fulfilled or (partially) violated

Config	Revenue model	Distance	Number of added: number of existing requests								
			4:8			6:6			8:4		
			Mixed	Neg	Pos	Mixed	Neg	Pos	Mixed	Neg	Pos
0.2/0.5	*EqOne*	Approx	36.4	0.0	63.7	54.9	0.0	45.1	70.3	0.0	29.7
		Exact	13.5	0.0	86.6	15.7	0.0	84.4	20.3	0.0	79.7
	*EqAll*	Approx	0.0	0.0	100.0	0.0	0.0	100.0	0.0	0.0	100.0
		Exact	0.0	0.0	100.0	0.0	0.0	100.0	0.0	0.0	100.0
	*DiffOne*	Approx	58.9	0.0	41.1	82.2	0.0	17.8	91.8	0.0	8.3
		Exact	38.7	0.0	61.3	59.3	0.0	40.8	72.6	0.0	27.5
	*DiffAll*	Approx	1.1	0.0	98.9	4.5	0.0	95.5	8.2	0.0	91.8
		Exact	0.0	0.0	100.0	0.0	0.0	100.0	0.0	0.0	100.0
0.2/1.0	*EqOne*	Approx	95.9	0.0	4.1	99.7	0.0	0.3	100.0	0.0	0.0
		Exact	61.5	0.0	38.6	76.7	0.0	23.3	83.8	0.0	16.3
	*EqAll*	Approx	11.4	0.0	88.6	17.2	0.0	82.8	37.1	0.0	62.9
		Exact	0.3	0.0	99.7	0.3	0.0	99.8	1.0	0.0	99.0
	*DiffOne*	Approx	96.9	0.0	3.2	99.9	0.0	0.1	100.0	0.0	0.1
		Exact	80.8	0.0	19.2	94.9	0.0	5.1	98.3	0.0	1.8
	*DiffAll*	Approx	50.9	0.0	49.1	80.5	0.0	19.6	94.3	0.0	5.7
		Exact	6.1	0.0	94.0	12.2	0.0	87.9	17.7	0.0	82.3
0.8/0.5	*EqOne*	Approx	45.8	5.9	48.4	65.1	0.7	34.2	82.3	0.0	17.7
		Exact	95.7	0.3	4.1	98.5	0.0	1.5	99.5	0.0	0.5
	*EqAll*	Approx	21.1	0.0	78.9	34.1	0.0	66.0	47.7	0.0	52.3
		Exact	42.8	0.0	57.3	52.9	0.0	47.1	50.8	0.0	49.2
	*DiffOne*	Approx	55.4	6.3	38.4	76.1	0.8	23.2	90.2	0.0	9.8
		Exact	98.2	1.2	0.6	99.9	0.1	0.0	100.0	0.0	0.0
	*DiffAll*	Approx	24.6	0.0	75.4	43.8	0.0	56.2	66.4	0.0	33.7
		Exact	79.0	0.0	21.1	92.4	0.0	7.6	92.2	0.0	7.8
0.8/1.0	*EqOne*	Approx	91.1	0.8	8.2	98.5	0.0	1.5	99.6	0.0	0.4
		Exact	92.6	0.1	7.4	97.4	0.0	2.7	98.5	0.0	1.5
	*EqAll*	Approx	51.9	0.0	48.1	60.7	0.0	39.3	69.2	0.0	30.8
		Exact	34.4	0.0	65.6	43.8	0.0	56.2	44.9	0.0	55.1
	*DiffOne*	Approx	91.6	2.5	5.9	98.9	0.0	1.2	99.9	0.0	0.1
		Exact	97.7	1.3	1.1	100.0	0.0	0.1	100.0	0.0	0.0
	*DiffAll*	Approx	82.3	0.0	17.8	94.4	0.0	5.7	97.9	0.0	2.1
		Exact	73.4	0.0	26.7	92.4	0.0	7.6	96.4	0.0	3.7

Neg: Adding a request decreases value for all bundles

Pos: Adding a request increases value for all bundles

Mixed: Adding a request increases value for some bundles and increases for others

We first consider monotonicity. Table 1 presents the analysis for monotonicity at the level of problems. Since we have already shown that when considering distances, a violation of monotonicity cannot occur, this table only refers to evaluations based on profits and considers the different revenue models. “Pos″ refers to cases in which adding a request to a bundle consistently increases marginal profit for all requests and all bundles. “Neg″ refers to the opposite case, and “Mixed″ is the fraction of problems in which both situations occur for some requests and bundles. As could be expected, situations in which monotonicity is consistently violated are rather rare but nevertheless still occur. In most cases (except for the configuration 0.8/0.5, in which existing requests are widely spread but added requests are more concentrated), the exact evaluation of tour lengths leads to fewer violations of monotonicity. Violations of monotonicity are less likely to occur if prices are high enough so that each bundle is profitable for all bidders (scenarios *EqAll* and *DiffAll*) rather than for at least one bidder (scenarios *EqOne* and *DiffOne*). There is no clear pattern concerning the difference between equal prices for all requests and differentiated prices. When the number of additional requests increases, there are more bundles, and consequently, it becomes more likely that at least adding one request to one bundle in a problem violates monotonicity.

**Table 2:** Logistic regression on monotonicity condition being fulfilled for a valuation vector

	Estimate	Std. error
(Intercept)	− 1.092***	0.018
More existing requests (4:8)	0.703***	0.015
More additional requests (8:4)	− 0.447***	0.016
Large area existing (configurations 0.8/x)	− 2.473***	0.015
Large area additional (configurations x/1.0)	− 1.760***	0.014
Exact distances	0.428***	0.013
Equal prices (scenarios *Eqxxx*)	1.389***	0.013
Profitable for all bidders (scenarios *xxxAll*)	2.976***	0.015

***p < 0.1%

To test the statistical significance of these differences, we performed a logistic regression on a binary variable indicating whether the monotonicity condition is fulfilled for a valuation vector or not. The results of this regression are shown in Table 2 and confirm our previous interpretation. Compared to the reference case of an equal number of existing and added requests, problems that have more existing requests fulfill monotonicity significantly more often, and problems with more added requests significantly less often. Both existing and added requests being spread over a larger area reduces the probability of fulfilling monotonicity, while exact calculation of distances, equal revenues for all requests, and revenues that make bundles profitable for all bidders all increase the probability of fulfilling monotonicity.

**Table 3:** Fraction of cases in which monotonicity condition is fulfilled or violated (in %)

Config	Revenue model	Scenario	Number of added: number of existing requests								
			4:8			6:6			8:4		
			Neg	Zero	Pos	Neg	Zero	Pos	Neg	Zero	Pos
0.2/0.5	*EqOne*	Approx	3.1	0.0	96.9	1.2	0.0	98.8	0.6	0.0	99.4
		Exact	1.0	0.0	99.0	0.2	0.0	99.8	0.1	0.0	99.9
	*EqAll*	Approx	0.0	0.0	100.0	0.0	0.0	100.0	0.0	0.0	100.0
		Exact	0.0	0.0	100.0	0.0	0.0	100.0	0.0	0.0	100.0
	*DiffOne*	Approx	6.7	0.4	92.9	3.7	0.1	96.2	2.2	0.0	97.8
		Exact	3.7	0.3	96.1	1.8	0.0	98.2	0.8	0.0	99.2
	*DiffAll*	Approx	0.1	0.0	99.9	0.0	0.0	99.9	0.0	0.0	100.0
		Exact	0.0	0.0	100.0	0.0	0.0	100.0	0.0	0.0	100.0
0.2/1.0	*EqOne*	Approx	19.3	0.0	80.7	11.0	0.0	89.0	6.3	0.0	93.7
		Exact	6.8	0.1	93.1	2.6	0.0	97.3	1.0	0.0	99.0
	*EqAll*	Approx	0.9	0.0	99.1	0.3	0.0	99.7	0.2	0.0	99.8
		Exact	0.0	0.0	100.0	0.0	0.0	100.0	0.0	0.0	100.0
	*DiffOne*	Approx	26.4	1.1	72.4	17.5	0.2	82.3	11.6	0.1	88.4
		Exact	14.3	1.3	84.4	8.2	0.3	91.5	4.6	0.1	95.4
	*DiffAll*	Approx	5.1	0.3	94.5	3.4	0.1	96.5	2.1	0.0	97.9
		Exact	0.3	0.2	99.5	0.1	0.1	99.8	0.0	0.0	99.9
0.8/0.5	*EqOne*	Approx	22.4	0.0	77.6	15.2	0.0	84.8	9.8	0.0	90.2
		Exact	32.5	1.4	66.1	24.3	0.6	75.1	16.5	0.2	83.3
	*EqAll*	Approx	3.0	0.0	97.0	2.2	0.0	97.8	1.5	0.0	98.5
		Exact	6.3	1.9	91.8	4.0	0.6	95.4	1.7	0.1	98.2
	*DiffOne*	Approx	31.5	1.8	66.7	24.2	1.5	74.3	19.4	0.6	80.1
		Exact	46.8	11.3	41.9	42.6	6.3	51.1	36.5	3.1	60.4
	*DiffAll*	Approx	4.5	0.4	95.1	4.5	0.2	95.2	3.8	0.1	96.1
		Exact	16.7	9.5	73.8	15.5	4.9	79.5	11.4	2.0	86.6
0.8/1.0	*EqOne*	Approx	26.7	0.0	73.3	18.1	0.0	81.9	11.3	0.0	88.7
		Exact	25.8	1.8	72.4	16.5	0.7	82.8	9.1	0.3	90.6
	*EqAll*	Approx	7.4	0.0	92.6	4.1	0.0	95.9	2.3	0.0	97.7
		Exact	3.6	1.8	94.6	1.8	0.6	97.6	0.7	0.2	99.1
	*DiffOne*	Approx	46.7	2.9	50.4	37.8	1.0	61.2	27.3	0.3	72.4
		Exact	43.7	11.9	44.4	37.0	6.3	56.7	29.1	2.8	68.1
	*DiffAll*	Approx	18.8	1.8	79.4	14.5	0.5	85.0	9.7	0.1	90.2
		Exact	12.3	9.5	78.2	10.9	5.2	83.9	7.4	2.1	90.4

Neg: Adding request decreases value

Zero: Value constant when adding request

Pos: Adding request increases value

Table 3 performs a similar analysis at the level of individual bundles, i.e., by considering each instance of adding one request to a bundle separately. These results differ in some aspects from the analysis at the problem level. The most striking difference is the effect of the number of additional requests. While increasing that number makes it more likely that at least one violation of monotonicity occurs in a problem, the likelihood that this occurs at one particular combination of bundle and additional request decreases. Problems in which a large number of requests are added also contain many cases in which monotonicity is fulfilled. Considering also the relatively high number of request/bundle combinations in which monotonicity is fulfilled, we thus can conclude that many bundles fulfill that condition even in problems where monotonicity violations occur. Another remarkable difference between the analysis at the level of bundles and entire problems is that the effect of calculating distances exactly vs. by approximation is not visible at the level of bundles but only at the aggregate level.

Next, we consider additivity. Since we assume that revenues are additive, we just have to consider distances. If distances are subadditive, then for any pricing scheme, profits must be superadditive, and vice versa. We again begin by considering entire valuations.

**Table 4:** Fractions of valuations with different forms of additivity (in %)

Config	Distance	Number of added: number of existing requests								
		4:8			6:6			8:4		
		Mixed	Neg	Pos	Mixed	Neg	Pos	Mixed	Neg	Pos
0.2/0.5	Approx	2.2	0.0	97.8	7.4	0.0	92.6	15.2	0.0	84.9
	Exact	0.0	0.0	100.0	0.0	0.0	100.0	0.0	0.0	100.0
0.2/1.0	Approx	61.8	0.1	38.2	87.4	0.0	12.7	96.4	0.0	3.6
	Exact	6.7	0.0	93.3	12.5	0.0	87.6	15.4	0.0	84.7
0.8/0.5	Approx	25.0	0.0	75.0	45.1	0.0	54.9	69.7	0.0	30.3
	Exact	68.7	0.2	31.2	91.7	0.0	8.3	93.7	0.0	6.4
0.8/1.0	Approx	88.3	0.2	11.6	97.2	0.0	2.9	99.3	0.0	0.7
	Exact	52.6	0.1	47.3	80.0	0.0	20.0	88.9	0.0	11.1

Neg: Superadditive distance for all pairs of bundles (negative synergy)

Pos: Subadditive distance for all pairs of bundles (positive synergy)

Mixed: Valuation contains positive as well as negative synergies

Results of this analysis are shown in Table 4. The column labels refer to the sign of the difference $v_i(S) +v_i(T) - v_i(S\cup T)$. “Neg” (negative), therefore, refers to cases of superadditive distances in which the (marginal) tour length of serving all requests of two bundles exceeds the sum of marginal tour lengths when the bundles are served individually and therefore represents negative synergies between the bundles. Similarly, “Pos” refers to positive synergies (i.e., savings resulting from serving two sets of requests together). “Mixed” indicates the fraction of valuations in which positive and negative synergies occur. Clearly, only a few valuations contain only negative synergies. In some settings, the fraction of valuations exhibiting only positive synergies is quite large, but there are also cases where it is relatively low. To study the effect of problem characteristics, we again performed a logistic regression (Table 5). Similar to monotonicity, a larger number of added requests and requests spread over a larger area decrease the probability that additivity is fulfilled. In contrast to monotonicity, additivity is more often violated if distances are calculated exactly.

**Table 5:** Logistic regression on additivity being fulfilled for a valuation vector

	Estimate	Std. error
(Intercept)	2.792***	0.034
More existing requests (4:8)	0.964***	0.030
More added requests (8:4)	− 0.479***	0.029
Large area existing (configurations 0.8/x)	− 2.892***	0.028
Large area additional (configurations x/1.0)	−2.012***	0.027
Exact distances	−1.029***	0.024

***p < 0.1%

**Table 6:** Fractions of different forms of additivity (in %)

Config	Distance	Number of added: number of existing requests								
		4:8			6:6			8:4		
		Neg	Zero	Pos	Neg	Zero	Pos	Neg	Zero	Pos
0.2/0.5	Approx	0.2	0.0	99.8	0.1	0.0	99.9	0.0	0.0	100.0
	Exact	0.0	0.0	100.0	0.0	0.0	100.0	0.0	0.0	100.0
0.2/1.0	Approx	13.7	0.0	86.3	8.9	0.0	91.1	5.9	0.0	94.1
	Exact	1.1	3.0	95.9	0.7	1.2	98.0	0.4	0.6	99.0
0.8/0.5	Approx	8.3	0.0	91.7	8.1	0.0	91.9	7.3	0.0	92.7
	Exact	30.5	36.3	33.2	36.8	17.6	45.6	33.0	6.0	61.1
0.8/1.0	Approx	45.4	0.0	54.6	37.0	0.0	63.0	25.2	0.0	74.8
	Exact	19.6	42.5	37.9	22.7	21.8	55.6	18.0	7.9	74.2

Neg: Superadditive distance (negative synergy)

Zero: Additive distance (no synergy)

Pos: Subadditive distance (positive synergy)

Table 6 analyzes the additivity of bundles of requests. In the first configuration, where additional requests are outside the area of existing requests, all valuations are subadditive when route lengths are calculated exactly, and also, with approximate tour lengths, superadditive valuations occur only very rarely. However, this picture changes significantly in the other configurations. In configuration 0.8/1.0, where there is a large overlap between the areas in which existing requests are located, and the locations of new requests, almost 20% of all pairs of requests exhibit negative synergies when distances are calculated exactly, and values increase between 25% and 45% for approximate distances. Typically, synergies become more likely if more requests are to be added. In three out of the four configurations, negative synergies are more likely if route length is only approximated, but if additional requests are concentrated in a part of the area of existing requests (configuration 0.8/0.5), the opposite can be observed.

Finally, we consider the SNC condition. At the level of entire valuations, that condition was rarely ever fulfilled. For most settings, it was not fulfilled in any of the valuation vectors we generated; the largest number across all settings was half a percent. We, therefore, do not report detailed results at the level of valuations.

**Table 7:** Fraction of pairs of bundles for which the SNC condition is fulfilled or violated (in %)

Config.	Revenue model	Distance	Number of added: number of existing requests					
			4:8		6:6		8:4	
			Violated	Fulfilled	Violated	Fulfilled	Violated	Fulfilled
0.2/0.5	Dist	Approx	18.4	81.6	25.5	74.5	27.6	72.4
		Exact	18.7	81.3	27.3	72.7	30.6	69.4
	*EqOne*	Approx	18.4	81.6	25.5	74.5	27.6	72.4
		Exact	18.7	81.3	27.1	72.9	30.3	69.7
	*EqAll*	Approx	18.4	81.6	25.5	74.5	27.6	72.4
		Exact	18.6	81.4	26.9	73.1	30.2	69.8
	*DiffOne*	Approx	18.8	81.2	26.8	73.2	29.2	70.8
		Exact	18.9	81.1	27.6	72.4	30.9	69.1
	*DiffAll*	Approx	18.8	81.2	26.7	73.3	29.1	70.9
		Exact	18.8	81.2	27.3	72.7	30.6	69.4
0.2/1.0	Dist	Approx	18.5	81.5	25.7	74.3	27.9	72.1
		Exact	18.5	81.5	26.9	73.1	30.1	69.9
	*EqOne*	Approx	18.5	81.5	25.7	74.3	27.9	72.1
		Exact	18.6	81.4	27.0	73.0	30.2	69.8
	*EqAll*	Approx	18.5	81.5	25.7	74.3	27.9	72.1
		Exact	18.4	81.6	26.7	73.3	29.9	70.1
	*DiffOne*	Approx	18.8	81.2	27.0	73.0	29.7	70.3
		Exact	18.9	81.1	27.7	72.3	31.0	69.0
	*DiffAll*	Approx	18.8	81.2	26.8	73.2	29.3	70.7
		Exact	18.6	81.4	27.1	72.9	30.5	69.5
0.8/0.5	Dist	Approx	7.4	92.6	16.5	83.5	22.2	77.8
		Exact	16.6	83.4	25.4	74.6	29.8	70.2
	*EqOne*	Approx	7.4	92.6	16.5	83.5	22.2	77.8
		Exact	16.7	83.3	25.5	74.5	29.8	70.2
	*EqAll*	Approx	7.4	92.6	16.5	83.5	22.2	77.8
		Exact	16.6	83.4	25.3	74.7	29.2	70.8
	*DiffOne*	Approx	7.4	92.6	16.5	83.5	22.5	77.5
		Exact	16.7	83.3	25.6	74.4	29.9	70.1
	*DiffAll*	Approx	7.4	92.6	16.7	83.3	23.4	76.6
		Exact	16.6	83.4	25.5	74.5	29.8	70.2
0.8/1.0	Dist	Approx	14.8	85.2	23.2	76.8	27.1	72.9
		Exact	16.2	83.8	25.2	74.8	29.6	70.4
	*EqOne*	Approx	14.8	85.2	23.2	76.8	27.1	72.9
		Exact	16.2	83.8	25.2	74.8	29.4	70.6
	*EqAll*	Approx	14.8	85.2	23.2	76.8	27.1	72.9
		Exact	16.1	83.9	24.5	75.5	28.5	71.5
	*DiffOne*	Approx	14.8	85.2	23.4	76.6	27.8	72.2
		Exact	16.2	83.8	25.4	74.6	30.0	70.0
	*DiffAll*	Approx	14.9	85.1	24.0	76.0	28.8	71.2
		Exact	16.2	83.8	25.3	74.7	29.8	70.2

As Table 7 shows, the condition is actually quite often fulfilled for individual pairs of bundles. As with the other properties, adding a larger number of requests slightly decreases the probability of fulfilling the SNC condition from over 80% to about 70%. There are hardly any clear influences of other problem characteristics.

### Consistency between carriers

Next, we analyze whether the structural properties of valuations are consistent between carriers. We compare whether adding an item to a bundle leads to a violation of monotonicity for both players or combining two bundles leads to, e.g., superadditivity for one carrier, but subadditivity for the other one.

**Fig. 3:**
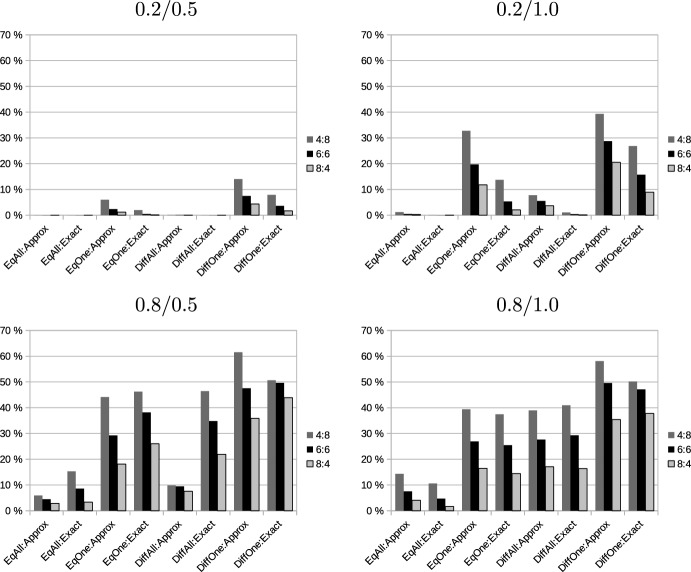
Occurrence of inconsistencies between carriers: Monotonicity

We first consider monotonicity. In this and the following analyses, we focus on violations of the respective properties. Figure 3 shows the fraction of cases in which adding a request to a bundle decreases the profit of the bundle under the different pricing models for one carrier but not for the other. Detailed data can be found in the online supplement. Typically, these fractions are larger than the fraction of monotonicity violations, so it seems very rare that monotonicity violations occur for both carriers simultaneously. There is a clear ranking of the different pricing scenarios. When we assume that requests are priced differently and profitable only for one carrier, violations of monotonicity are both most frequent (as demonstrated in Table 3) and also most differently distributed among players. We also observe that they occur more often in situations where only a small number of additional requests is added to a larger number of existing requests. In most cases, approximation of distances leads to a higher inconsistency, but there are also cases in which the level of inconsistency is about the same (in configuration 0.8/1.0) and also cases in which it is considerably higher for exact distances (pricing model *DiffAll* in configuration 0.8/0.5).

**Fig. 4:**
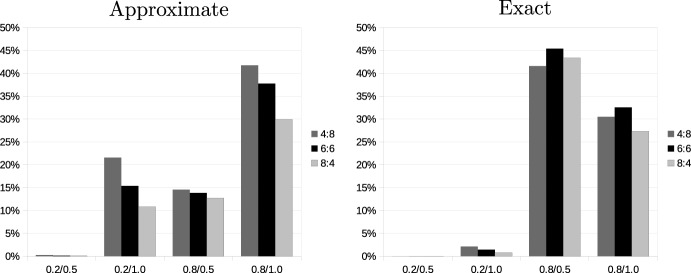
Occurrence of inconsistencies between carriers: Additivity

Figure 4 performs a similar analysis for additivity. We again consider only distances since revenues are additive. We observe considerable differences if distances are calculated exactly or approximated. For exact distances, the highest level of inconsistencies occurs in configuration 0.8/0.5, which exhibits only a moderate level of inconsistencies when distances are approximated. Furthermore, considering the number of requests with exact distances, most inconsistencies occur when 6 requests are added to 6 existing requests, while for approximate distances, the level of inconsistency monotonically decreases when the number of existing requests decreases.

**Fig. 5:**
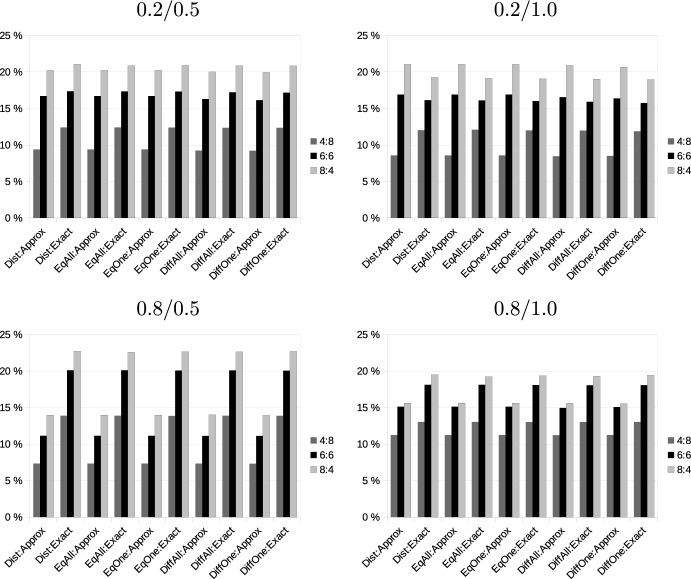
Occurrence of inconsistencies between carriers: SNC condition

Figure 5 shows the results for the SNC condition. As could already be seen from the previous results, this condition is mainly influenced by the number of added requests. This also holds for inconsistencies. The level of inconsistencies between carriers reaches about 20% in all problem settings when 8 additional requests are added to 4 existing requests. Apart from this effect, results are remarkably stable across different revenue models and ways of calculating distances. The only exception is scenario 0.8/0.5, in which relatively narrowly distributed additional requests are added to existing requests in a larger area. Here, the way of calculating distances has a strong effect, exact distances lead to considerably fewer inconsistencies between the valuations of players than approximate distances.

### Consistency between exact evaluation and approximation

The results presented above already indicate considerable differences between the exact and approximate calculation of distances. We further elaborate on this question in the following subsection. Concerning inconsistencies between the evaluations of different carriers, it was not relevant whether an inconsistency occurred because, e.g., carrier 1 has positive synergies between two bundles and carrier 2 has negative synergies or vice versa. The situation is different when we compare the exact and approximate calculations of the optimal tour length. The exact calculation provides the tour lengths and costs that will actually be encountered if the request (or bundle of requests) is assigned to and executed by the carrier. If the approximate calculation indicates a different relationship between requests, that might lead to wrong bids and distorted outcomes of the allocation mechanism. We, therefore, distinguish the two types of different evaluations and refer to the case in which the exact model indicates that a property is violated, while the approximation indicates that it is fulfilled as a type 1 deviation and the opposite case as a type 2 deviation.

**Fig. 6:**
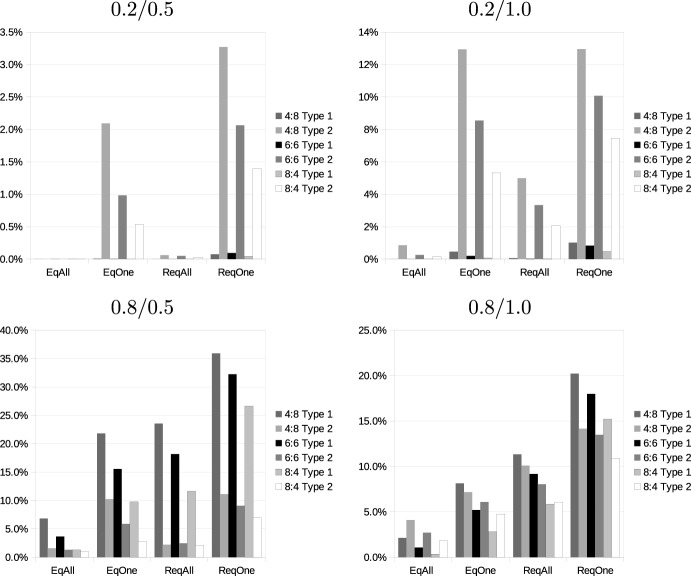
Inconsistency across distance calculations: Monotonicity

Figure 6 shows the frequency of occurrence of the different types of deviations in the evaluation of monotonicity. Details (including how often both methods for calculating tour lengths agree that adding a request to a bundle fulfills or violates monotonicity) can be found in the online supplement. The distribution of different types of deviations depends on the problem structure. In problems in which the carriers’ existing requests are highly concentrated, type 2 deviations far outnumber the occurrence of type 1, so here the approximation of tour lengths is more likely to indicate a violation of monotonicity than when they are determined exactly. If existing requests are distributed over a large part of the total area, we observe the opposite: the exact calculation of tour lengths more often indicates violations of monotonicity, and the number of violations is higher.

**Fig. 7:**
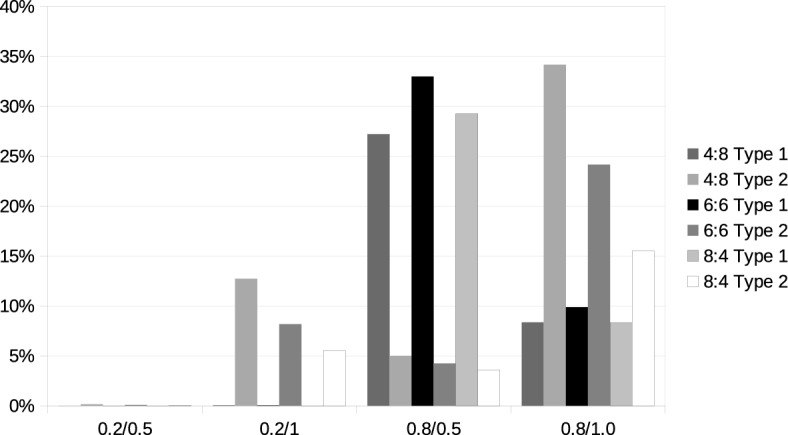
Inconsistency across distance calculations: Additivity

Figure 7 performs a similar analysis for additivity; details can again be found in the online supplement. Here we also find considerable differences between the geographical configurations. Changing the spread of additional requests from the central region in scenario 0.8/0.5 to the entire region in scenario 0.8/1.0 reverts the relationship between the two models. In the former case, the exact calculation of tour lengths is much more likely to indicate the presence of subadditivity; in the latter case, it is the approximate calculation.

**Fig. 8:**
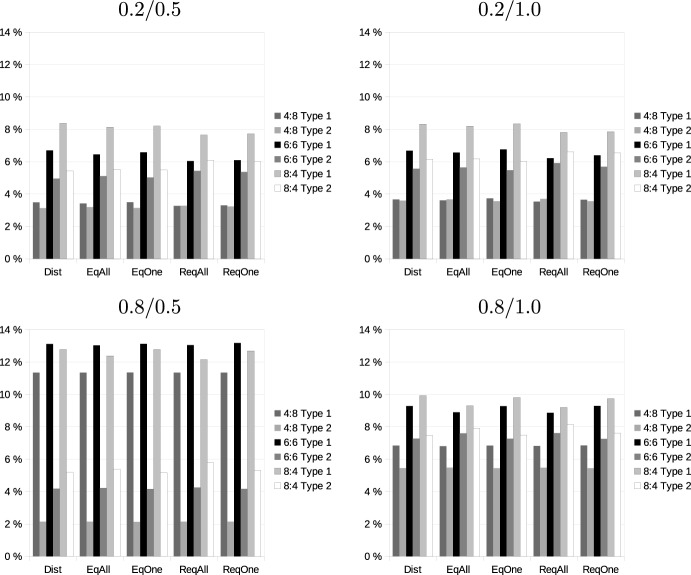
Inconsistency across distance calculations: SNC condition

Finally, Fig. 8 presents the analysis for the SNC conditions. Typically, across all settings, there are more type 1 than type 2 deviations. However, this difference is not large except for scenario 0.8/0.5. Inconsistency increases in the number of requests to be added but at a decreasing rate.

## Discussion and conclusions

Modern auction theory has identified a number of conditions on bidders’ valuations that allow for the use of efficient, innovative auction mechanisms. In the present paper, we have studied the question of whether valuations derived from a simple routing problem fulfill these conditions.

The answer to our research question has direct implications for the development of methods to support collaboration among carriers. Transportation problems are difficult to solve. If it is possible to develop auction mechanisms that require only a few evaluations of transportation problems, that would considerably simplify the task of bidders. Unfortunately, our results are not very encouraging. They clearly support the widespread notion that linear pricing (i.e., a pricing scheme in which each request is priced separately and the price of a bundle is simply the sum of the prices of all requests in the bundle) is not possible. We have found that the SNC condition is almost never completely fulfilled.

In fact, we found that if one considers the entire vector of valuations of a carrier, one frequently finds at least some violations, even of fundamental requirements such as monotonicity or superadditivity. However, when taking a closer look, one also finds that although some violations might exist in each bidder’s valuations, large parts of these valuations actually fulfill these properties, and only a few bundles (or pairs of bundles) lead to violations. A challenge for auction theory could thus be to develop robust mechanisms, which work well if valuations “almost” fulfill properties such as superadditivity or similar conditions or to quantify the impact of not-too-frequent violations on the auction outcomes.

Another result that could strongly influence the design of collaboration mechanisms is the impact of the way of calculating distances. We have shown that using approximations of tour length might change the structural properties of the resulting valuation vector considerably. Thus, the approach suggested in the literature to use simple approximation formulas instead of actually solving routing problems could have undesirable side effects on the functioning of auction mechanisms in which carriers bid based on these approximations. This insight could add a new topic to research on tour length approximations: A good approximation method should not only provide a precise estimate of actual tour lengths but should also be able to reproduce the structural properties of the true valuation vector.

Concerning our last research questions, our results indicate that problem characteristics such as the number of existing requests and requests to be added, as well as their geographical location, can have a considerable impact on whether conditions such as monotonicity or superadditivity are fulfilled or not. In contrast, it seems that the SNC condition is less influenced by these factors.

The research we have presented in this paper is thus only a starting point. It would be interesting to perform similar studies for more realistic transportation problems and involve a larger number of carriers with more realistic distributions of both existing and additional (exchanged) requests. Considering a larger number of carriers would, e.g., allow us to determine if there are clusters of carriers whose valuations share common properties. Such studies, in particular, if they are based on real data from carriers, could offer more insight into which auction mechanisms are appropriate for carrier collaboration and which are not.

### Supplementary Information

Below is the link to the electronic supplementary material.

Supplementary file1 (PDF 73 kb)

## Footnotes

1 Valuation functions are said to be supermodular if $v_i(S_1)+v_i(S_2)\le v_i(S_1\cup S_2)+v_i(S_1\cap S_2)$.

2 Note that $$\left( \begin{array}{c}n\\ l\end{array}\right) = \left( \begin{array}{c}n\\ n-l\end{array}\right)$$.
