# Supplementary material for: Properties of bundle valuations in carrier collaboration
Source: Cent Eur J Oper Res. 2023 Jul 26;32(2):241–66. doi: 10.1007/s10100-023-00873-1 (PMC11032298; doi:10.1007/s10100-023-00873-1)
Supplement: Supplementary file 1 — Supplementary file1 (PDF 73 kb) [file 10100_2023_873_MOESM1_ESM.pdf]

Online supplement to

Properties of bundle valuations in carrier collaboration

R. Vetschera, D. Knyazev, D. Rehsmann

	Number of added : number of existing requests								
	4:8			6:6			8:4		
	Both viol.	Different	Both fullf.	Both viol.	Different	Both fullf.	Both viol.	Different	Both fullf.
Configuration: 0.2/0.5									
Approx	0.0	0.3	99.7	0.0	0.1	99.9	0.0	0.1	99.9
Exact	0.0	0.0	100.0	0.0	0.0	100.0	0.0	0.0	100.0
Configuration: 0.2/1.0									
Approx	2.9	21.6	75.5	1.2	15.4	83.4	0.5	10.8	88.7
Exact	0.0	2.1	97.9	0.0	1.5	98.5	0.0	0.8	99.2
Configuration: 0.8/0.5									
Approx	1.0	14.6	84.4	1.1	13.8	85.0	1.0	12.7	86.3
Exact	9.7	41.6	48.7	14.1	45.4	40.5	11.3	43.4	45.3
Configuration: 0.8/1.0									
Approx	24.5	41.8	33.7	18.1	37.8	44.2	10.2	29.9	59.9
Exact	4.4	30.5	65.2	6.4	32.6	61.1	4.3	27.3	68.4

Both viol: Additivity violated for both bidders

Different: Additivity violated for one bidder only

Both fullf: Additivity fulfilled for both bidders

Table 1: Consistency of additivity across bidders (in %)

		Number of added : number of existing requests								
		4:8			6:6			8:4		
		Both viol.	Different	Both fullf.	Both viol.	Different	Both fullf.	Both viol.	Different	Both fullf.
Configuration: 0.2/0.5										
EqAll	Approx	0.0	0.0	100.0	0.0	0.0	100.0	0.0	0.0	100.0
	Exact	0.0	0.0	100.0	0.0	0.0	100.0	0.0	0.0	100.0
EqOne	Approx	0.1	6.0	93.9	0.0	2.4	97.6	0.0	1.2	98.8
	Exact	0.0	1.9	98.1	0.0	0.4	99.6	0.0	0.1	99.9
DiffAll	Approx	0.0	0.1	99.9	0.0	0.1	99.9	0.0	0.0	100.0
	Exact	0.0	0.0	100.0	0.0	0.0	100.0	0.0	0.0	100.0
DiffOne	Approx	0.1	14.0	85.9	0.1	7.4	92.5	0.0	4.3	95.6
	Exact	0.0	7.9	92.1	0.0	3.6	96.4	0.0	1.7	98.3
Configuration: 0.2/1.0										
EqAll	Approx	0.3	1.2	98.5	0.0	0.5	99.5	0.0	0.3	99.7
	Exact	0.0	0.0	100.0	0.0	0.0	100.0	0.0	0.0	100.0
EqOne	Approx	2.9	32.8	64.3	1.2	19.7	79.2	0.4	11.8	87.8
	Exact	0.0	13.7	86.3	0.0	5.3	94.7	0.0	2.1	97.9
DiffAll	Approx	1.6	7.8	90.7	0.7	5.5	93.7	0.3	3.7	96.1
	Exact	0.0	1.1	98.9	0.0	0.4	99.6	0.0	0.1	99.9
DiffOne	Approx	7.9	39.3	52.8	3.3	28.7	67.9	1.4	20.5	78.1
	Exact	2.2	26.9	71.0	0.6	15.7	83.7	0.2	8.9	90.9
Configuration: 0.8/0.5										
EqAll	Approx	0.0	5.9	94.1	0.0	4.5	95.5	0.0	2.9	97.1
	Exact	0.6	15.3	84.2	0.3	8.6	91.1	0.1	3.3	96.5
EqOne	Approx	0.3	44.1	55.6	0.6	29.2	70.2	0.7	18.1	81.2
	Exact	10.8	46.2	42.9	5.8	38.2	56.0	3.7	26.0	70.3
DiffAll	Approx	0.0	9.8	90.2	0.0	9.5	90.5	0.1	7.6	92.4
	Exact	3.0	46.4	50.6	3.1	34.8	62.1	2.4	21.9	75.7
DiffOne	Approx	2.5	61.5	35.9	1.9	47.5	50.5	2.0	35.9	62.1
	Exact	32.8	50.6	16.6	24.1	49.6	26.3	17.6	43.9	38.5
Configuration: 0.2/0.5										
EqAll	Approx	0.2	14.4	85.4	0.3	7.5	92.2	0.3	4.1	95.6
	Exact	0.1	10.6	89.3	0.1	4.7	95.2	0.0	1.7	98.3
EqOne	Approx	6.9	39.4	53.6	4.6	26.9	68.5	3.1	16.5	80.5
	Exact	8.9	37.4	53.7	4.5	25.4	70.1	2.2	14.4	83.4
DiffAll	Approx	1.1	39.0	59.9	1.2	27.6	71.2	1.2	17.1	81.7
	Exact	1.3	41.0	57.7	1.5	29.3	69.2	1.4	16.4	82.3
DiffOne	Approx	20.5	58.1	21.4	14.0	49.6	36.4	9.9	35.4	54.7
	Exact	30.6	50.2	19.3	19.8	47.1	33.1	13.0	37.8	49.2

Both viol: Monotonicity violated for both bidders
Different: Monotonicity violated for one bidder only
Both fullf: Monotonicity fulfilled for both bidders

Table 2: Consistency of monotonicity across bidders (in %)

		Number of added : number of existing requests								
		4:8			6:6			8:4		
Revenue	Distance	Both viol.	Different	Both fullf.	Both viol.	Different	Both fullf.	Both viol.	Different	Both fullf.
Configuration: 0.2/0.5										
Dist	Approx	13.7	9.4	76.9	17.2	16.7	66.1	17.5	20.2	62.3
	Exact	12.5	12.4	75.1	18.6	17.4	64.1	20.0	21.1	58.9
EqAll	Approx	13.7	9.4	76.9	17.2	16.7	66.1	17.5	20.2	62.3
	Exact	12.4	12.4	75.2	18.2	17.3	64.5	19.8	20.9	59.3
EqOne	Approx	13.7	9.4	76.9	17.2	16.7	66.1	17.5	20.2	62.3
	Exact	12.5	12.4	75.1	18.4	17.3	64.3	19.9	20.9	59.2
DiffAll	Approx	14.2	9.2	76.6	18.5	16.3	65.2	19.1	20.0	60.9
	Exact	12.6	12.4	75.0	18.7	17.2	64.1	20.2	20.9	58.9
DiffOne	Approx	14.2	9.2	76.6	18.8	16.1	65.1	19.2	19.9	60.8
	Exact	12.7	12.4	75.0	19.0	17.2	63.9	20.5	20.8	58.7
Configuration: 0.2/1.0										
Dist	Approx	14.2	8.6	77.2	17.3	16.9	65.8	17.4	21.1	61.6
	Exact	12.5	12.0	75.5	18.8	16.1	65.1	20.5	19.3	60.3
EqAll	Approx	14.2	8.6	77.2	17.3	16.9	65.8	17.4	21.1	61.6
	Exact	12.4	12.1	75.5	18.6	16.1	65.3	20.4	19.1	60.5
EqOne	Approx	14.2	8.6	77.2	17.3	16.9	65.8	17.4	21.1	61.6
	Exact	12.7	12.0	75.4	19.0	16.0	65.0	20.7	19.1	60.2
DiffAll	Approx	14.6	8.5	77.0	18.6	16.6	64.9	18.9	20.9	60.2
	Exact	12.6	12.0	75.4	19.2	15.9	64.9	21.0	19.0	59.9
DiffOne	Approx	14.5	8.5	77.0	18.8	16.4	64.8	19.3	20.7	60.0
	Exact	12.9	11.9	75.2	19.8	15.7	64.4	21.5	19.0	59.5
Configuration: 0.8/0.5										
Dist	Approx	3.8	7.3	88.9	10.9	11.1	77.9	15.2	14.0	70.8
	Exact	9.7	13.9	76.4	15.4	20.1	64.5	18.4	22.7	58.9
EqAll	Approx	3.8	7.3	88.9	10.9	11.1	77.9	15.2	14.0	70.8
	Exact	9.7	13.9	76.4	15.3	20.1	64.6	17.9	22.6	59.5
EqOne	Approx	3.8	7.3	88.9	10.9	11.1	77.9	15.2	14.0	70.8
	Exact	9.7	13.9	76.4	15.4	20.1	64.5	18.5	22.7	58.9
DiffAll	Approx	3.8	7.3	88.9	11.1	11.1	77.8	16.4	14.0	69.6
	Exact	9.7	13.9	76.4	15.4	20.1	64.5	18.5	22.6	58.9
DiffOne	Approx	3.8	7.3	88.9	11.0	11.1	77.9	15.6	13.9	70.5
	Exact	9.7	13.9	76.4	15.5	20.1	64.4	18.6	22.7	58.7
Configuration: 0.8/1.0										
Dist	Approx	9.2	11.2	79.6	15.6	15.1	69.3	19.3	15.6	65.1
	Exact	9.7	13.0	77.3	16.1	18.1	65.8	19.8	19.5	60.7
EqAll	Approx	9.2	11.2	79.6	15.6	15.1	69.3	19.3	15.6	65.1
	Exact	9.6	13.0	77.3	15.4	18.1	66.5	18.9	19.2	61.9
EqOne	Approx	9.2	11.2	79.6	15.6	15.1	69.3	19.3	15.6	65.1
	Exact	9.7	13.0	77.3	16.1	18.1	65.8	19.7	19.4	60.9
DiffAll	Approx	9.3	11.2	79.5	16.5	14.9	68.5	21.0	15.6	63.4
	Exact	9.7	13.0	77.3	16.2	18.1	65.7	20.2	19.3	60.5
DiffOne	Approx	9.2	11.2	79.5	15.8	15.1	69.1	20.1	15.6	64.4
	Exact	9.7	13.0	77.2	16.4	18.1	65.6	20.3	19.4	60.3

Both viol: SNC condition violated for both bidders

Different: SNC condition violated for one bidder only

Both fullf: SNC condition fulfilled for both bidders

Table 3: Consistency of SNC condition across bidders (in %)

		Number of added : number of existing requests					
		4:8		6:6		8:4	
				Exact			
Config	Approx	Viol.	Fullf.	Viol.	Fullf.	Viol.	Fullf.
0.2/0.5	Viol.	0.0	0.2	0.0	0.1	0.0	0.0
	Fullf.	0.0	99.8	0.0	99.9	0.0	100.0
0.2/1.0	Viol.	1.0	12.7	0.7	8.2	0.4	5.5
	Fullf.	0.0	86.2	0.0	91.1	0.0	94.1
0.8/0.5	Viol.	3.3	5.0	3.8	4.2	3.7	3.6
	Fullf.	27.2	64.5	33.0	59.0	29.2	63.4
0.8/1.0	Viol.	11.2	34.2	12.8	24.2	9.7	15.5
	Fullf.	8.4	46.2	9.9	53.2	8.3	66.5

Rows: Additivity violated (Viol.) / fulfilled (Fullf.) for approximate distances
Columns: Additivity violated (Viol.) / fulfilled (Fullf.) for exact distances

Table 4: Consistency of additivity across distance calculation methods (in %)

			Number of added : number of existing requests					
			4:8		6:6		8:4	
					Exact			
Config	Revenue	Approx	Viol.	Fullf.	Viol.	Fullf.	Viol.	Fullf.
0.2/0.5	EqAll	Viol.	0.0	0.0	0.0	0.0	0.0	0.0
		Fullf.	0.0	100.0	0.0	100.0	0.0	100.0
	EqOne	Viol.	1.0	2.1	0.2	1.0	0.1	0.5
		Fullf.	0.0	96.9	0.0	98.8	0.0	99.4
	ReqAll	Viol.	0.0	0.1	0.0	0.1	0.0	0.0
		Fullf.	0.0	99.9	0.0	99.9	0.0	100.0
	ReqOne	Viol.	3.9	3.3	1.7	2.1	0.8	1.4
		Fullf.	0.1	92.8	0.1	96.1	0.0	97.8
0.2/1.0	EqAll	Viol.	0.0	0.9	0.0	0.3	0.0	0.2
		Fullf.	0.0	99.1	0.0	99.7	0.0	99.8
	EqOne	Viol.	6.4	12.9	2.5	8.5	1.0	5.3
		Fullf.	0.5	80.2	0.2	88.8	0.1	93.6
	ReqAll	Viol.	0.5	5.0	0.2	3.3	0.1	2.1
		Fullf.	0.1	94.5	0.0	96.5	0.0	97.9
	ReqOne	Viol.	14.6	13.0	7.6	10.1	4.2	7.5
		Fullf.	1.0	71.4	0.8	81.5	0.5	87.9
0.8/0.5	EqAll	Viol.	1.4	1.6	0.9	1.3	0.5	1.0
		Fullf.	6.8	90.2	3.7	94.1	1.3	97.2
	EqOne	Viol.	12.1	10.3	9.3	5.9	6.9	2.8
		Fullf.	21.8	55.8	15.6	69.2	9.8	80.5
	ReqAll	Viol.	2.7	2.2	2.3	2.5	1.7	2.1
		Fullf.	23.6	71.5	18.2	77.1	11.6	84.5
	ReqOne	Viol.	22.2	11.1	16.6	9.1	12.9	7.0
		Fullf.	35.9	30.8	32.3	42.0	26.6	53.4
0.8/1.0	EqAll	Viol.	3.3	4.1	1.4	2.7	0.5	1.8
		Fullf.	2.1	90.5	1.1	94.8	0.4	97.3
	EqOne	Viol.	19.5	7.2	12.0	6.1	6.5	4.8
		Fullf.	8.1	65.2	5.2	76.7	2.8	85.9
	ReqAll	Viol.	10.5	10.1	6.9	8.1	3.7	6.1
		Fullf.	11.3	68.1	9.2	75.8	5.9	84.4
	ReqOne	Viol.	35.4	14.2	25.3	13.5	16.7	10.9
		Fullf.	20.2	30.2	18.0	43.2	15.2	57.2

Rows: Monotonicity violated (Viol.) / fulfilled (Fullf.) for approximate distances
Columns: Monotonicity violated (Viol.) / fulfilled (Fullf.) for exact distances

Table 5: Consistency of monotonicity across distance calculation methods (in %)

			Number of added : number of existing requests					
			4:8		6:6		8:4	
					Exact			
Config	Revenue	Approx	Viol.	Fullf.	Viol.	Fullf.	Viol.	Fullf.
0.2/0.5	Dist	Viol.	15.2	3.1	20.6	5.0	22.2	5.4
		Fullf.	3.5	78.1	6.7	67.8	8.4	64.0
	EqAll	Viol.	15.2	3.2	20.4	5.1	22.1	5.5
		Fullf.	3.4	78.2	6.4	68.0	8.1	64.2
	EqOne	Viol.	15.2	3.1	20.5	5.0	22.1	5.5
		Fullf.	3.5	78.1	6.6	67.9	8.2	64.2
	ReqAll	Viol.	15.5	3.3	21.2	5.4	23.0	6.1
		Fullf.	3.3	78.0	6.0	67.3	7.6	63.3
	ReqOne	Viol.	15.6	3.2	21.5	5.4	23.2	6.0
		Fullf.	3.3	77.9	6.1	67.1	7.7	63.1
0.2/1.0	Dist	Viol.	14.9	3.6	20.2	5.6	21.8	6.1
		Fullf.	3.7	77.9	6.7	67.6	8.3	63.8
	EqAll	Viol.	14.8	3.7	20.1	5.6	21.7	6.2
		Fullf.	3.6	77.9	6.6	67.7	8.2	63.9
	EqOne	Viol.	14.9	3.6	20.3	5.5	21.9	6.0
		Fullf.	3.7	77.8	6.8	67.5	8.3	63.8
	ReqAll	Viol.	15.1	3.7	20.9	5.9	22.7	6.6
		Fullf.	3.5	77.7	6.2	66.9	7.8	62.8
	ReqOne	Viol.	15.2	3.6	21.3	5.7	23.1	6.5
		Fullf.	3.7	77.6	6.4	66.6	7.9	62.5
0.8/0.5	Dist	Viol.	5.3	2.1	12.3	4.2	17.0	5.2
		Fullf.	11.4	81.2	13.1	70.4	12.8	65.0
	EqAll	Viol.	5.3	2.1	12.3	4.2	16.8	5.4
		Fullf.	11.4	81.2	13.0	70.5	12.4	65.4
	EqOne	Viol.	5.3	2.1	12.3	4.2	17.0	5.2
		Fullf.	11.4	81.2	13.1	70.4	12.8	65.0
	ReqAll	Viol.	5.3	2.1	12.4	4.3	17.6	5.8
		Fullf.	11.4	81.2	13.0	70.3	12.2	64.4
	ReqOne	Viol.	5.3	2.1	12.4	4.2	17.2	5.3
		Fullf.	11.4	81.2	13.2	70.3	12.7	64.8
0.8/1.0	Dist	Viol.	9.4	5.5	15.9	7.3	19.7	7.5
		Fullf.	6.8	78.3	9.3	67.6	9.9	63.0
	EqAll	Viol.	9.3	5.5	15.6	7.6	19.2	7.9
		Fullf.	6.8	78.4	8.9	67.9	9.3	63.6
	EqOne	Viol.	9.4	5.4	15.9	7.3	19.6	7.5
		Fullf.	6.8	78.3	9.3	67.6	9.8	63.1
	ReqAll	Viol.	9.4	5.5	16.4	7.6	20.6	8.1
		Fullf.	6.8	78.3	8.9	67.1	9.2	62.0
	ReqOne	Viol.	9.4	5.4	16.1	7.3	20.2	7.6
		Fullf.	6.9	78.3	9.3	67.3	9.7	62.4

Rows: SNC condition violated (Viol.) / fulfilled (Fullf.) for approximate distances
Columns: SNC condition violated (Viol.)/ fulfilled (Fullf.) for exact distances

Table 6: Consistency of SNC condition across distance calcuation methods (in %)
